# Mechanism of fungal remediation of wetland water: *Stropharia rugosoannulata* as promising fungal species for the development of biofilters to remove clinically important pathogenic and antibiotic resistant bacteria in contaminated water

**DOI:** 10.3389/fmicb.2023.1234586

**Published:** 2023-10-18

**Authors:** Keya Sen, Marina Llewellyn, Babak Taheri, Robert J. Turner, Tanner Berglund, Kellen Maloney

**Affiliations:** ^1^Division of Biological Sciences, School of Science, Technology, Engineering and Mathematics (STEM), University of Washington, Bothell, WA, United States; ^2^School of Interdisciplinary Arts and Sciences, University of Washington, Bothell, WA, United States

**Keywords:** fungal remediation, mycelia, antibiotic resistance, biofilter, fungus-bacteria interaction

## Abstract

Mycoremediation uses mushroom forming fungi for remediation of sites contaminated with biotic and abiotic contaminants. The root-like hyphae of many fungi, the mycelia, have been used to remediate soil and water. In this study mushroom mycelia biofilters were evaluated for remediation efficacy of wetland water polluted with crow feces containing antibiotic resistant (AMR) bacteria. Three strains of fungi, *Pleurotus ostreatus, Stropharia rugosoannulata*, and *Pleurotus pulmonarius*, were allowed to develop dense mycelia for 3-5 weeks on wood chips within cylindrical jars. Biofilter jars were incubated with wetland water (WW) obtained from a crow roost area that was additionally spiked with AMR bacteria isolated from previous crow fecal collections. *E. coli, Staphylococcus aureus, Enterococcus faecium, Campylobacter jejuni, Klebsiella pneumoniae, Pseudomonas aeruginosa*, and *Salmonella enteritidis* were added at concentrations of 1,500–3,500 CFU/100 ml. Remediation was calculated from bacterial counts or gene copy numbers (GCN), before and after passage of water through jars. *Stropharia* and *P. pulmonarius* biofilters remediated all bacteria, but *Klebsiella*, in the range of 43-78%, after 1 h. Incubation of water for 24 h showed *Stropharia* remediation to be superior relative to other tested fungi. Percent remediation varied as follows: *S. aureus*-100%, E. *faecium*-97%, *C. jejuni*-59%, *P. aeruginosa*-54%, *E. coli*-65% and *S. enteritidis*-27%. The mechanism of remediation was tested by removing the mycelium from the biofilter column after passage of water, followed by extraction of DNA. Association of bacterial DNA with the mycelia was demonstrated by qPCR for all bacteria, except *S. aureus* and *Salmonella*. Depending on the bacteria, the GCN ranged from 3,500 to 54,000/250 mg of mycelia. Thus, some of the ways in which mycelia biofilters decrease bacteria from water are through bio-filtration and bio-absorption. Active fungal growth and close contact with bacteria appear necessary for removal. Overall these results suggest that mushroom mycelia biofilters have the potential to effectively remediate water contaminated with pathogenic and AMR bacteria.

## 1. Introduction

Wetlands and streams within urban watersheds provide various ecological benefits to mankind, including water quality enhancement. However, these critical areas are often vulnerable to bacterial pollution by birds, resident wild animals, and stormwater drainage. Many of these bacteria may be pathogenic- and antibiotic-resistant, becoming a threat to human health as well as to the environment because of their ability to survive or even proliferate in the water for long periods (Leclerc et al., 2002; Gaffield et al., 2003; Ahmed et al., 2019). Treatment strategies for removing pathogens from surface waters are usually conducted in engineered systems (e.g., bioretention cells). Such strategies involve damage by exposure to ultraviolet radiation via sunlight or to reactive oxygen species (Khaengraeng and Reed, 2005); killing by antimicrobial (AM) activity of root exudates (Axelrood et al., 1996); trapping within biofilms formed on the surface or within the water (Jasper et al., 2013); physical removal of pathogens using biofilters or biofilms made on surfaces such as sand, gravel, or rocks (Beutel and Larson, 2015; Maurya et al., 2020); removal by sedimentation of particles to which pathogens have attached; and predation and natural mortality after sedimentation and/or trapping of pathogens by nematodes, protozoa, and rotifers (Green et al., 1997; Decamp and Warren, 1998). These practices, while eco-friendly and relatively cheap to install, do not consistently remove pathogens to levels that are required for primary or secondary contact recreation activities in receiving waters (Clary et al., 2008). Furthermore, there is a need to develop self-sustaining, natural treatment processes that will remove pathogens from polluted waters specific to an urban setting (Jasper et al., 2013).

The use of fungi in the removal of *E. coli* and fecal coliform, where the mycelium of the fungus was intentionally cultivated to serve as bioreactors for bioremediation of water bodies, was first demonstrated in 2005 (Thomas et al., 2009). An experiment completed in 2009 involved the passing of lagoon waste through a rain garden inoculated with two species of fungi (Thomas et al., 2009; Taylor et al., 2015). When the cells were infused with lagoon waste containing a high concentration of fecal coliforms (259,000 CFU/100 ml), the treatment cell demonstrated an outflow below 10 CFU/100 ml, which was much less than the control cell at 376 CFU/100 ml. Recently, Pini and Geddes showed that *P. ostreatus*-inoculated straw was able to remove *E. coli* in the laboratory and simulated field sampling-based settings at 99.25 and 99.74%, respectively, over a period of 96 h (Pini and Geddes, 2020).

The resiliency of the mycoremediation system is an important factor in scaling experiments to real-world applications. A test of the most resilient fungus and filter material combinations to be used in mycofilters, so that they retained their biological activity under simulated stressful environmental conditions, was reported by Beutel and Larson (2015). The study demonstrated that a substrate of mixed wood chips and straw colonized with mycelium from *Stropharia rugosoannulata* or *Irpex lacteus* was the most promising among the eight fungal strains and five substrate combinations. However, these studies only reported the removal of the indicator organism *E. coli* and fecal coliforms. These indicators may not always be the best organism to indicate the presence and subsequent removal of avian-borne pathogens such as *Enterococcus faecium, Campylobacter jejuni, Salmonella* species, and other non-fermenting Gram-negative organisms (Benskin et al., 2009; Wu et al., 2009). In surface wetlands, a 1-log removal (90% remediation) has been reported for fecal coliforms (Vymazal, 2005; Kadlec and Wallace, 2008), while for actual pathogens, efficiencies up to 2-log have been reported (Jasper et al., 2013). Thus, it is important to test the removal of specific pathogens.

We reported earlier that a constructed wetland within the University of Washington Bothell/Cascadia College Campus contains a variety of pathogenic and antibiotic-resistant bacteria, with many of them being multidrug-resistant (Sen et al., 2019, 2022). Multi-locus sequence typing of the antibiotic-resistant *E. coli* and *Campylobacter* strains indicated wild birds as a primary source for these bacteria. Our goal was to test whether mycoremediation could reduce the bacterial load and the presence of antibiotic-resistant genes in the Bothell wetlands. Three species of lignin-degrading fungi were used to inoculate wood chips and create biofilter material. They were *Pleurotus ostreatus var florida*, an environmental species of *Pleurotus pulmonarius*, and *Stropharia rugosoannulata* (*Stropharia*). Biofilters were inoculated with two types of water: crow feces-contaminated wetland water and crow feces-contaminated wetland water (WW) spiked with seven clinically significant bacteria that have been shown to be carried by birds: *E. coli, Klebsiella pneumoniae, C. jejuni, Salmonella* species, *Pseudomonas aeruginosa, Staphylococcus aureus*, and *Enterococcus faecium* (Benskin et al., 2009). All of these bacterial species were found in the Bothell wetland water or in the crow feces within the last 5 years. Remediation of 10 antimicrobial resistance (AMR) genes, *bla*_CTX_, *bla*_CMY_, *sul-1, tet* (A), *tet* (B), *strB* and *strA, tet* (M), *tet* (O), and *van*A, was additionally studied; these had been shown to be present in the wetlands (Roberts et al., 2016; Sen et al., 2019, 2022). Finally, a potential mechanism of fungal remediation of bacteria, that of the capture of bacteria by the mycelia, was examined.

## 2. Materials and methods

### 2.1. Bacterial strains and growth conditions

The *E. coli* strains F11.2 and F42.2 were obtained from crow feces in 2014. They have been described earlier (Sen et al., 2019; Table S1). F11.2 carries antibiotic resistance genes: *bla*_CTX_, *tet* (M), *tet* (A), *strA*, and *sul1*. F42.2 carries the *bla*_CMY_ and *str*B genes. Strain F42.2 was grown on Luria Broth (LB) containing ampicillin (50 μg/ml) and F11.2 on tetracycline (16 μg/ml) and cefotaxime (4 μg/ml). *C. jejuni* strain F39.2, a fecal isolate, has been described earlier and carries the *tet* (O) resistance gene (21). Other strains: *S. enterica* subsp. *enteritidis* ATCC 13076, *Enterococcus. faecium Van*^*R*^ ATCC 700221, *P. aeruginosa* ATCC 27583, *K. pneumoniae*, ATCC BA 2146, and *S. aureus* ATCC 25923, were obtained from the American Type Culture Collection (ATCC). *S. enteritidis* was maintained on xylose lysine deoxycholate (XLD) plates, *P. aeruginosa* on m-PA agar, *S. aureus* on mannitol salt and CHROMagar™ Staph aureus (Hardy Diagnostics), and *E. faecium* on bile esculin or blood agar (Table S1).

### 2.2. Preparation of biofilter material

Sterile Red Alder (*Alnus rubra*) wood chips were prepared by soaking overnight in 1% hydrogen peroxide, followed by sterilization in an autoclave at 120°C for 15 min. *Pleurotus ostreatus var. florida* and *Stropharia rugosoannulata* spawn plugs were obtained from Fungi Perfecti Inc., WA. They were maintained on 2% malt extract agar plates and further expanded on hydrated and sterile rye berry (*Secale cereale*) seeds purchased from Amazon.com. Once the rye berry seeds were fully colonized with mushroom mycelia, they were layered onto the sterilized wood chips. The material was grown at 30°C for 3–5 weeks. An environmental *Pleurotus* species was obtained from Denny Park, Kirkland, WA, and the identity was verified by fungal ITS gene sequencing to be *P. pulmonarius*. The sequence of the 679 bp product, obtained from the ITS locus in the genomic DNA of the isolate using ITS1 and ITS4 primers, was submitted to GenBank and has the accession number ON5957991. A second verification was performed using primers LiO3 and L3 that targeted the D1/D2 region of the large subunit of the ribosomal RNA gene (Eberhardt, 2012). This set was not as specific, but the 473 bp product obtained showed 100% homology to several species within the Pleurotus genus, including *P. pulmonarius*.

DNA extraction is described below, and the primers used in the PCR reaction are presented in Table S2.

### 2.3. Preparation of biofiltration columns and bottles

Columns were used for the measurement of single time points (Figure S1A). For experiments where water had to be retained in the columns and agitated for different lengths of time, 12-oz glass jars were used. They were filled with alternate layers of mycelium-covered rye berry seed and wood chips. The wood chips were allowed to colonize for 3–5 weeks at 30°C to form a compact column inside the bottle (Figure S1B, Frame A). The bottles were thus substituted for the columns. Water had to be physically removed from the bottles and the water could not percolate and come out from any opening in the bottom as in the columns.

### 2.4. Preparation of spiked water

Overnight cultures of *E. coli, S. enteritidis, S. aureus*, and *K. pneumoniae*, grown in TSY broth, *E. faecium* grown in Brain Heart infusion, and 48-hour culture of Campylobacter grown on CVA blood agar were used to spike the water. The wetland water was spiked with ~1500–3500 CFU/100 ml of each bacteria. The exact spiked number was determined by plating 10 μl of a 10^4^ CFU/ml dilution of the overnight culture on appropriate selective plates for each species of bacteria.

### 2.5. Collection of wetland water

Two liters of water were collected in sterile bottles from the site RS2 within the wetland of the University of Washington, Bothell. The map of the wetland and the location within the wetland can be found in an earlier study (Sen et al., 2019). Briefly, this site borders the crow roost area, with the run-off water originating from the campus that flows through a bioswale prior to discharging at this site. This water tends to have fairly uniform counts of *E. coli* bacteria, with a median value of 196 CFU/100 ml over 40 samples collected between 2011 and 2021. Mean values for other water quality parameters measured periodically at this site between 2007 and 2021 are as follows: pH = 7.3, conductivity = 282 μS, nitrate = 0.45 mg/l, turbidity = 3.7 NTU, salinity = 0.2 ppm. Temperatures were between 12°C and 16°C. Variability in these parameters at this site is relatively low, and there is no apparent seasonality to the recorded values, though rainfall tends to slightly increase both turbidity and nitrate levels at the sampling site. The water used for this study was collected during a dry period.

### 2.6. Biofiltration and bioincubation

Field samples of contaminated wetland water were added to 12-oz Mason jars containing wood chips previously colonized with mycelia. The wetland water was added directly in some experiments or spiked with seven different bacteria described above (Figures S1B, S2). One bottle was used exclusively for each time point (Figure S2). Outflow water was collected at specified time intervals by physically pouring out the water samples and analyzing them as described below. After biofiltration or bioincubation, 2.5 ml of water was collected in triplicate, passed through a 0.45-μm membrane filter, and analyzed for *E. coli* and *S. aureus* by selective plating, while 45 ml was poured out and membrane filtered for DNA analysis. The filters were stored at −20°C until ready for DNA extraction. A schematic representation of the process is shown in Figure S2.

### 2.7. Extraction of DNA from mycelia and qPCR

*S. rugosoannulata* mycelia-covered wood chips were removed from a biofilter column that had either been used for the remediation of spiked wetland water or from a control column that was incubated with only sterile distilled water. Approximately 250 mg of mycelia were carefully scraped from the wood chips with a blade and collected in a microfuge tube. A Qiagen QIAamp^®^ PowerFecal^®^ Pro DNA Kit, which had earlier proved successful in extracting DNA from crow fecal samples, was used to extract the total DNA from the isolated mycelia. The protocol outlined in the Qiagen handbook was followed with minor modifications. Beads for homogenization of the samples were transferred into the microfuge tubes that contained the weighed mycelia (instead of the samples being placed into the power bead tubes). After the addition of CD1 lysis buffer, the samples were incubated for 10 min at 65°C. The rest of the procedure was followed exactly as described by the manufacturer, and the final elution volume was 50 μl. The control column was scraped out and extracted in a separate facility so that there was no possibility of contamination between the two column extractions. GCN of each of the seven bacteria present in the DNA extracts of mycelia from the control or spiked water biofilter column were obtained by performing TaqMan qPCR with appropriate primers and probes as described in Table S2. Gene copy number (GCN) was calculated from standard curves developed as described below.

### 2.8. qPCR and enumeration in gene copy numbers

For the quantification of the reduction in *C. jejuni, P. aeruginosa, E. faecium, K. pneumoniae*, and *S. enteritidis* and antibiotic resistance genes, the GCN was measured in the water samples by TaqMan qPCR. Total DNA was extracted directly from the 0.45 μm filters using a Qiagen DNEasy Power water kit (Qiagen Inc.). Prior to extraction, each filter was spiked with an *E. coli* Bioball (Biomerieux Inc., https://www.biomerieux-usa.com) that contained 1,000 copies of *E. coli* containing the single-copy plasmid HPY3. The plasmid can be detected using a specific primer and probe set and used as a sample processing control (SPC); its construction has been described (Sen et al., 2007). Samples with a two-quantification cycle (Cq) delay in the SPC were considered to have potential PCR inhibitors or sub-optimal extraction and were removed from measurements of remediation. qPCR was performed using the appropriate primers and probes (Table S2) targeted to the antibiotic resistance genes, namely, tetracycline resistance genes: *tet* (A), *tet* (B), *tet* (M), and *tet* (O); streptomycin-resistant genes: *strA* and *strB*; sulfonamide resistance gene: *sul1*; beta-lactamase genes: *bla*_CMY_ and *bla*_CTX_; and vancomycin resistance gene: *van*A or to the seven bacterial spp. Controls and standards were generated for quantitative measurements of each of the genes using PCR amplicons that were cloned into *E. coli* cells (Topo^®^ TA Cloning Kit, Invitrogen) or used directly (Sen et al., 2019). For all seven bacteria, the whole genome was used to make the standards and subsequent standard curves.

### 2.9. Calculation of remediation by CFU and gene copy number (GCN) and Statistical analysis

When measurements were made in CFU, the CFU obtained before and after passage through the biofilter column were incorporated in the following equation:


$$\begin{array}{l} \text{\% Remediation in CFU}\\ =\frac{\text{CFU in spiked water}-\text{CFU in spiked water post biofilter}}{\text{CFU in spiked water}}\times 100 \end{array}$$

When measurements were made in GCN, remediation was calculated as follows:


$$\begin{array}{l} \text{\% Remediation in GCN}\\ =\frac{\text{GCN in spiked water}-\text{GCN in spiked water post biofilter}}{\text{GCN in spiked water}}\times 100 \end{array}$$

The precision of the calculation of the percent remediation at each time point was obtained by calculating the standard deviation from three replicates.

## 3. Results

### 3.1. Occurrence of bacteria in wetland water

Bacteria occurring in the wetland water were measured at least three times during 2020–2021 (Table 1). Wherever possible, bacterial cells were recovered and cultured on appropriate selective plates. Thus, *E.coli, S. aureus*, and *P. aeruginosa* were routinely cultivated from the water, while we had more confidence in our qPCR estimation for the other bacteria. As expected, *E. coli, C. jejuni*, and *E. faecium* were consistently found in abundance in the water. *P. aeruginosa, K. pneumoniae*, and *S. aureus* were the least abundant but were always detected (Table 1). *Salmonella* species could be confirmed only twice using qPCR for the *inv*A gene.

**Table 1:** Bacteria present in 100 ml of wetland water.

**Bacterial species**	**12/3/2020**	**4/1/2021**	**7/12/2021**	**7/21/2021**	**9/23/2021**
**(A)**					
*E. coli* (CFU)	170	ND	180	115	432
*P. aeruginosa* (CFU)	ND	ND	70	56	152
*S. aureus* (CFU)	ND	160	30	208	20
**(B)**					
*E. faecium* (GCN)	ND	21,395	ND	128	37,174
*C. jejuni* (GCN)	22,278	5,013,440	ND	1,344,114	59,875
*K. pneumoniae* (GCN)	ND	ND	ND	460	121

ND indicates not determined. GCN indicates gene copy numbers/per 100 ml of water, while CFU indicates colony-forming units/100 ml of water.

### 3.2. Comparison of three biofilters for the remediation of seven bacterial species in wetland water after 1-h and 20-h of residence in the jars

**Figure 1:**
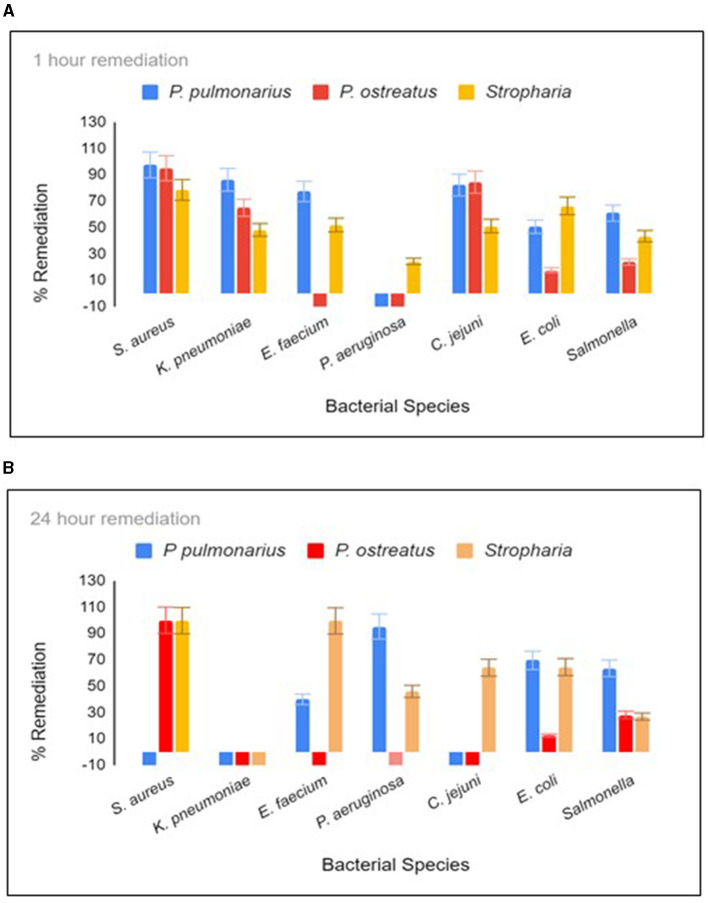
Remediation after 1 h **(A)** and 20 h **(B)** residence in the biofilter jar. Each time point was tested in triplicate. For *C. jejuni, E. faecium, K. pneumoniae*, and *P. aeruginosa*, remediation was measured using qPCR, and the absolute GCN was obtained from a standard curve generated from chromosomal DNA dilutions (10,000 to 10 gene copies) for each respective bacterial species. The colony forming unit (CFU) of *S. aureus* and *E. coli* were measured by counting the mauve colonies on CHROM agar Staph Aureus (Hardy diagnostics) and blue colonies on Mcoli agar plates, respectively. *Stropharia* indicates the fungus species *Stropharia rugosoannulata*.

Three different biofilter materials were used in three respective 12-oz Mason jars (Figure S1B), and they were woodchips colonized with either *P. ostreatus, P. pulmonarius*, or *S. rugosoannulata*. The biofilters were allowed to develop in the jar for no more than 4 weeks, during which time there was dense colonization of all three fungal species within the jar. *E. coli* and *S. aureus* were calculated by counting the CFU on m-ColiBlue and CHROMagarSA selective plates, respectively. TaqMan PCR was performed to obtain the GCN of the remaining bacteria present in the water sample. When the spiked water was held in the jar for 1 h with constant agitation on a shaker at room temperature, the *S. rugosoannulata* column proved to be the most successful in terms of remediation of the maximum species of bacteria in 1 h (Figure 1A). Incubation was continued for 24 h when *S. rugosoannulata* showed remediation for all species except *K. pneumoniae* (Figure 1B). Growth of *K. pneumoniae* was observed in all three types of biofilters after 20 h (Figure 1B). Percentage remediation by *P. pulmonarius* proved to be superior to *S. rugosoannulata* in the first hour for five of the bacteria, reaching 97.9, 86.5, 77.4, 82.2, and 60.9% for *S. aureus, K. pneumoniae, E. faecium, C. jejuni*, and *S. enteritidis*, respectively, while for *E. coli*, almost similar remediation was achieved by either of the biofilters (50.6 and 66.4%).

### 3.3. Remediation of antibiotic-resistant genes

**Figure 2:**
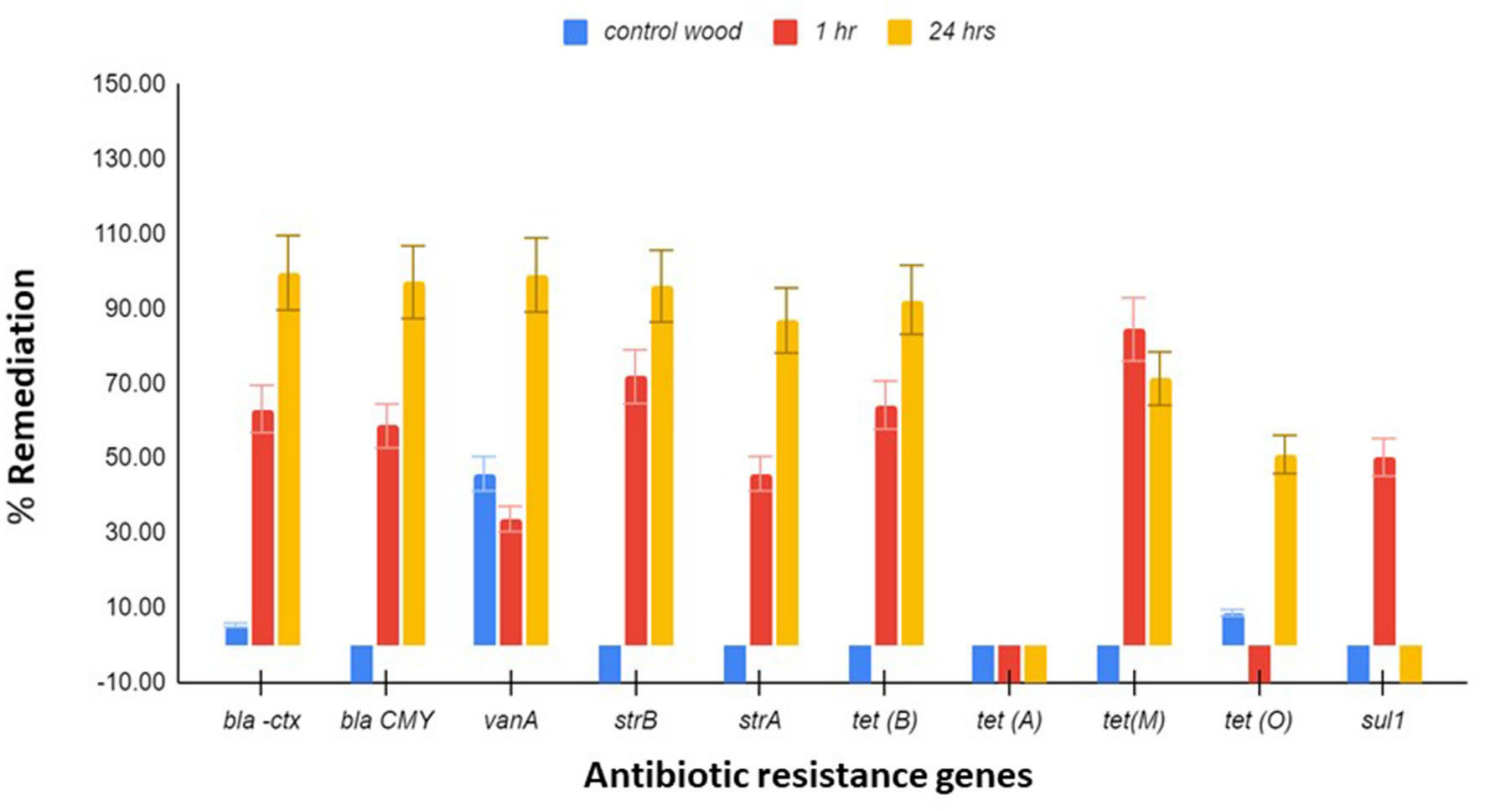
Antibiotic resistance (AR) gene remediation. Triplicate water samples of 45 ml each were removed from the jar and membrane-filtered at 1 h and 24 h, and the extracted DNA was used for determining the remediation of *bla*_CTX_, *bla*_CMY_, *vanA, sul-1, strB, strA, tet* (A), *tet* (B), *tet* (M), and *tet* (O) by qPCR. Control wood had no mycelia. Flowchart of the process is presented in Figure S2. The results presented are averages of three different experiments, and the standard error bars are indicated.

Ten antibiotic-resistant genes, which were earlier shown to be present in the wetland, were tested for remediation by the *Stropharia* biofilters using the same DNA extracts used to study the remediation of bacteria. Since other species of bacteria present in the water could have contributed to the pool of AR genes, this measurement was necessary. After 24 h of residence time in a biofilter, *bla*_CTX_, *bla*_CMY_, *vanA, strB, strA*, and *tet* (B) showed remediation of 99.6, 97.11, 99.04, 96.08, 86.88, and 92.41%, respectively (Figure 2). Both *tet* (M) and *sul* 1 were best remediated at 1 h, with a reduction of 84.4 and 50.1%, respectively (Figure 2). Remediation of *tet* (O) appeared to be dependent on the remediation of *C. jejuni*, as demonstrated by regression analysis (adjusted *R*^2^ = 0.38, coefficient = 11.7, *p* = 0.0188), and the remediation of *vanA* on the remediation of *E. faecium* (adjusted *R*^2^ = 0.48, coefficient = 1.37, and *p* = 0.016). Remediation of *tet* (B) was shown to be dependent on the remediation of *E. coli* (adjusted *R*^2^ = 0.46, coefficient = 1.08, *p* = 0.0396), as was *tet* (M) (adjusted *R*^2^ = 0.298; coefficient = 0.614752, *p* = 0.03834).

### 3.4. Capture of bacteria by mycelia

Biofilters in jars were incubated for 1 h with either sterile water, which served as a control, or WW spiked with five species of bacteria (Table 2). Scraped mycelia from the biofilter columns were extracted for DNA using the Qiagen QIAamp PowerFecal Pro DNA kit. An additional heating step greatly increased the yield from 250 mg of mycelia, which was the upper limit of extraction, and additional mycelia introduced PCR inhibitors. Since the mycelium of the fungus usually permeates the substrate, creating an extensive network together with the substrate, our DNA extraction included some wood substrate along with mycelia. When tested using qPCR, the incorporation of six bacteria into the mycelia was seen, with values ranging from ~3700 GCN (*Pseudomonas*) to ~78,000 GCN (*Campylobacter*; Table 2 and Figure S3). *S. aureus* and *Campylobacter*, which were not additionally spiked but tested from the background water, showed very different capture patterns, with *S. aureus* displaying no capture while *Campylobacter* had the maximum capture among all bacteria (Figure S3). *Pseudomonas* capture appeared to be low in all three trials; however, capture was definitely displayed. Capture of *Salmonella* was not demonstrated, but the bacteria were present in the background mycelia.

**Table 2:** Capture of bacteria by *Stropharia* mycelia.

**Bacteria**	**Sterile water (GCN)**	**Spiked water (GCN)**	**Result**
*Pseudomonas*	2106.27	5778.02	Capture
S. aureus^*^	99.00	119.00	No capture
*E. faecium*	5042.38	27712.24	Capture
*Campylobacter* spp^*^	5537.00	83395.50	Capture
*E. coli*	744.62	10637.17	Capture
*K. pneumoniae*	2487.00	31123.33	Capture
*Salmonella*	1280.00	1310.00	No capture

GCN is the gene copy number present in 250 mg of mycelia.

* WW is not spiked with *S. aureus* or *Campylobacter* spp. and the GCN is contributed by bacteria present in background water.

## 4. Discussion

The use of mycoremediation for clean-up of recalcitrant dyes, pesticides, pharmaceutically active compounds, and other micropollutants, including antibiotics and AMR genes, is increasingly being reported because they form an eco-friendly and economical way of remediation (Harms et al., 2011; Lucas et al., 2016; Castellet-Rovira et al., 2018; Hultberg et al., 2020). The non-specific ligninolytic enzymatic system of many fungi, including those of basidiomycetes *Pleurotus* and *Stropharia*, the intricate and extensive hyphal network that develops vigorously under optimal nutrient conditions, and the ability of many of them to adapt to fluctuating pH and temperature and develop resistance to heavy metals make them well suited for remediation of contaminated matrices such as soil and water (Harms et al., 2011). Although the diameter of the hyphae is small, between 2 and 10 μm, they can extend between 10^2^ and 10^4^ m per g depending on the type of topsoil and have been reported to cover a surface area of 3–90 m^2^/m^2^ of the upper 10 cm (3.95 in.) of soils (Taylor et al., 2015). This effectively increases the surface area-to-volume ratio and the subsequent range of remediation activities. The growth phase of the fungi has been shown to be important in the successful remediation of chemicals and organic micropollutants (Lucas et al., 2016; Hultberg et al., 2020); an actively growing fungus has hyphae that is constantly lengthening, secreting abundant exoenzymes at its tips, and contributing to the development of the intricate mycelial network in the process (Harms et al., 2011). Our study showed that the growth state of fungi was equally important in pathogen removal, and a fungal species with a well-developed and dense network of mycelia was more efficient than one that was still developing, one that was past its prime and was dying, or one that had entered the fruiting body formation stage. *Stropharia mycelia* developed for 4–5 weeks proved to be superior at remediation in terms of the maximum number of species of bacteria, while *Pleurotus* species needed only 3 weeks to develop dense mycelia for the remediation of six of the seven bacteria tested in the 1-h time frame. *Pleurotus* species are thus superior in terms of the rapidity with which they develop mycelia, as observed earlier (Hultberg et al., 2020), while *Stropharia* have a broader remediation effect that includes both Gram-positive and Gram-negative bacteria.

In attempting to remediate waterborne pathogens in a water body, three things were taken into consideration. The first was that mycelium had to be firmly attached to a substrate, such as wood chips, so that flowing water did not tear away the mycelium. Our initial attempt with straw resulted in the disintegration of the biofilter material after 1 h (results not shown). Second, the matrix on which the mycelium was to be cultivated should prevent the growth of molds. Several failures were encountered, where the mycelium stopped colonizing the substrate after the first couple of weeks due to mold developing on the substrate in the jars. Thus, pasteurization of the wood chips, which served as the substrate, was necessary. Immersing the wood chips in 0.3–1% hydrogen peroxide for at least 24 h proved to be effective. Since agitation has been shown to greatly increase the remediation of dyes by certain species of mushrooms (Sani et al., 1998), we introduced agitation to our biofilter columns in our wetland water trials. For *E. coli*, we achieved 51–66% remediation in 1 h, compared to 26 and 30% reported in other studies (Taylor et al., 2015). Incubation or retention of the contaminated water with the biofilters increased remediation compared to the other studies where the water was allowed to pass through the column at a rate of 0.5 L/min (Taylor et al., 2015). Retention or slow flow rates have been shown to increase remediation of pathogens in biofilters comprised of sand particles amended with mature biofilms by allowing interaction between pathogens and biofilms (Maurya et al., 2020) as well as in constructed treatment wetlands (Vymazal, 2005). In this study, retention time probably increases the interaction between pathogens and the fungal mycelia and their secreted enzymes. However, the possibility of the regrowth of bacteria such as *Klebsiella* and other total coliforms remain. *K. pneumoniae* and *Salmonella* showed renewed growth after initial remediation of ~50% in this study. Fecal coliform bacteria have been shown to regrow 100-fold in stored gray water in 24–48 h (Rose et al., 1991; Dixon et al., 2000). In this study, the wetland water was responsible for this since the wood was pretreated with peroxide and then sterilized. The dissolved solids in the wetland water may have played a role; our total dissolved solids (TDS) measurements were usually in the range of 250–300 ppm, which is considered in the low range by the U.S. EPA. Studies have also shown that *Campylobacter* and *Salmonella* die off in the presence of other competing bacteria (Ottoson and Stenström, 2003). However, in our background of wetland water, *C. jejuni* did show growth in the *Pleurotus* columns after 20 h, after showing an initial decrease. The conditions created in these jars may have led to microaerophilic niches, leading to the increase in *C. jejuni*. *Stropharia* biofilters were adequate in the removal of 56% of the bacteria, which was the average of three trials. A lot of variation was seen in the presence of *Campylobacter* in the wetland water; as a result, the remediation of *Campylobacter* was variable. In one of the trials, there was an actual increase in the number within 24 h. Although we ensured the presence of the seven bacteria that were tested by spiking the water with a fixed number of bacteria, we could not control the exact number of bacteria present in the background water on a given day.

The exact mechanism by which the fungal mycelia inactivates the bacteria is a matter of ongoing investigation. An important finding of this study was that five of the seven species tested are adsorbed, attached, or entrapped in the *Stropharia* mycelia from our background of wetland water. The GCN incorporated varied on the number of bacteria present in the background water as expected, and the greater the number present the higher the observed capture. We were initially surprised at repeatedly finding bacterial genes in mycelium biofilters that were only incubated with sterile distilled water (control biofilters). Since sterile conditions were maintained throughout, including the preparation of biofilters, which involved sterilizing the wood chips and the rye berry seeds as well, the bacteria could have come only from the fungi. Indeed, qPCR detected bacterial genes in mycelia isolated from pure cultures of all three fungi that were grown in Petri dishes of malt agar (results not shown). Many different species of fungi can host a diverse range of bacterial symbionts in their hyphae in an endosymbiotic relationship (Hoffman and Arnold, 2010). These symbiotic bacteria within the fungal mycelia can affect their host in a variety of ways, such as encouraging hyphal growth and providing or changing environmental pH (Frey-Klett et al., 2011). Bacteria have also been seen to rapidly move through the mycelium of fungi over long distances (Frey-Klett et al., 2011). Since mycelium-capturing bacteria seem to be a widespread trait among a diverse range of fungi for a broad range of functions, one possible mechanism may be that *S. rugosoannulata* removes the bacteria from the water by physically capturing the bacteria within the hyphae of their mycelium. This may come in the form of intra-hyphal colonization or simple planktonic physical association (Frey-Klett et al., 2011). Additionally, biodegradation via the secondary metabolites secreted by the hyphae may play a role in decreasing bacterial numbers (Fermor and Wood, 1981; Grant et al., 1984; Barron, 1988). The mycelial exudates exhibited different effects on different bacteria when the turbidity of the bacteria was measured in the presence of the exudates; however, the maximum effect appears to be in the first 6 h (unpublished results).

It would be inaccurate to estimate the remediation of AMR genes achieved by the fungal biofilters based on the remediation of the parent bacteria with which the water was spiked with alone. Many of them could be coming from unknown bacteria present in the background wetland water due to stormwater overflows and other pollution events. Moreover, resistance genes such as *tet* (A), *strA, strB*, and *sul1*, which are known to be present on plasmids containing transposons, could easily move to other bacteria by horizontal gene transfer. We have shown mobility of the *bla*_CMY_, *tet* (A), and *bla*_CTX_ from F42.2 and F11.2 earlier in our conjugation studies in the laboratory (Sen et al., 2020). Thus, it became important to test and quantitate them directly from DNA extracted from the filters using TaqMan qPCR. A significant correlation was seen between the remediation of *tet* (O) and *C. jejuni* (*p* = 0.0188), *van*A and *E. faecium* (*p* = 0.016), *tet* (B) and *E. coli* (*p* = 0.0396), and *tet* (M) and *E. coli* (*p* = 0.03834), indicating that these genes did not originate from other species of bacteria that may have been present in the wetland. Regression analysis for *bla*_CTX_, *bla*_CMY_, *sul-1, tet* (A), *strB*, and *strA* did not show dependence on the remediation of *E. coli*, even though *E. coli* strains containing these AMR genes were used as spikes. The lack of correlation could in part be attributed to the comparison of measurements of live cell counts, used for *E. coli*, against gene copy counts that were used for all AMR genes; the latter could come from live as well as dead cells.

## 5. Conclusion

In conclusion, we have optimized the use of *Stropharia* biofilters for the removal or reduction of seven clinically relevant bacterial species in a wetland polluted by crow feces. Using a prototype of a glass jar containing wood chips amended with *Stropharia* mycelia, we have demonstrated that agitation and increased holding time can reduce four bacterial species by >95% and others by at least 48% from starting concentrations of 3 × 10^3^−10^4^ CFU/100 ml when water is incubated in these jars. Of the 10 AMR genes tested, although the dynamics of remediation were different between the genes, by 24 h, seven of the genes were remediated by >80% and one by ~50% while two showed increased presence. Although incubation for 24 h achieves maximum remediation for five of the seven bacteria tested, 1 h of incubation would be preferred when testing actual field samples since two of the bacteria can regrow in 24 h. The wetland water polluted by crow feces had a pH of ~7.5 in most of the water collections, and although the temperature of the water varied from the spring to the summer months by ~4°C (12–16°C), bacteria are not expected to grow rapidly in this temperature range. Testing of water from different sources for remediation of their bacterial content by the fungal biofilters would further help in evaluating the effectiveness of these filters under different water quality parameters. Furthermore, metagenome analysis conducted with DNA acquired from these water samples before and after passage through biofilters would provide information about the remediation of a wide variety of bacteria in addition to the seven bacteria tested above.

## Data availability statement

The raw data supporting the conclusions of this article will be made available by the authors, without undue reservation.

## Author contributions

KS conceptualized and supervised all microbiology and qPCR-based experiments and analysis, performed some experiments, performed data analysis, and prepared the manuscript. KM conceptualized the fungal biofilters, prepared them, and performed the initial biofiltration studies evaluating indicator organisms. ML prepared the biofilters, collected water and feces samples, and performed many of the microbiology and qPCR-based experiments, as well as data analysis. BT and TB collected samples, determined AMR using phenotypic and genotypic methods, and performed qPCR and data analysis. RT identified sites in the wetland, evaluated the remediation of indicator organisms, obtained funding, and helped with the writing of the manuscript. All authors contributed to the article and approved the submitted version.
