# Supplementary material for: Mechanism of fungal remediation of wetland water: Stropharia rugosoannulata as promising fungal species for the development of biofilters to remove clinically important pathogenic and antibiotic resistant bacteria in contaminated water
Source: Front Microbiol. 2023 Oct 18;14:1234586. doi: 10.3389/fmicb.2023.1234586 (PMC10642173; doi:10.3389/fmicb.2023.1234586)
Supplement: Supplementary file 1 [file Data_Sheet_1.pdf]

Mechanism of Fungal remediation of wetland water: *Stropharia rugosoannulata* as promising fungal species for the development of biofilter to remove clinically important pathogenic and antibiotic resistant bacteria in contaminated water

Supplemental Figures and Tables

A

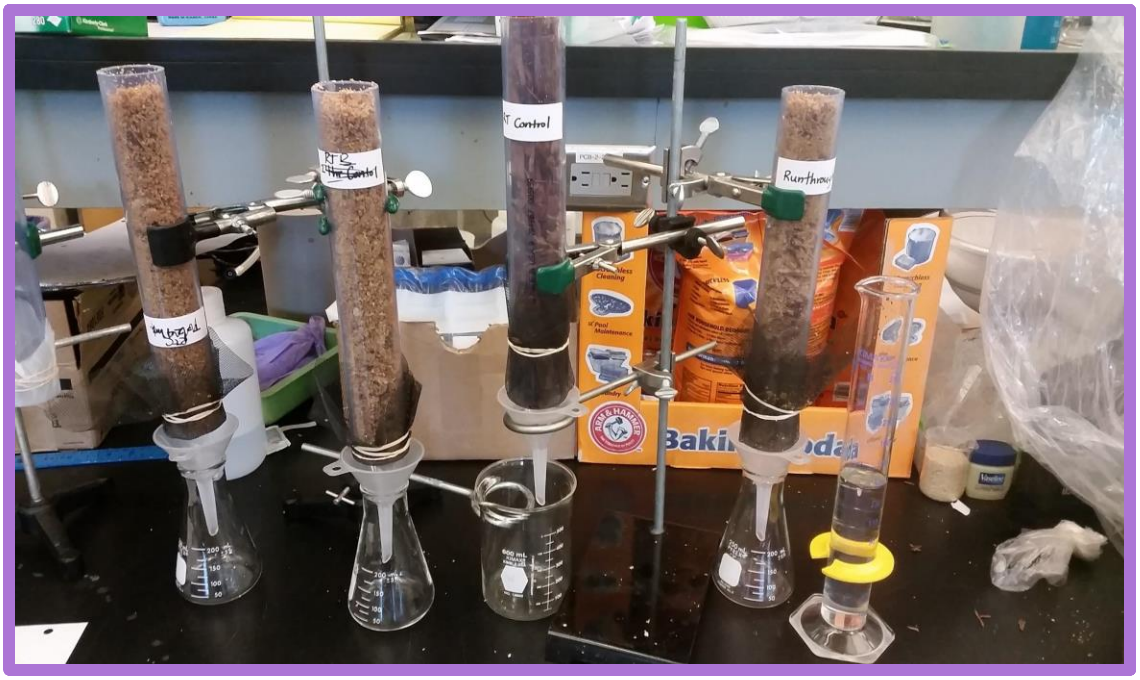

B

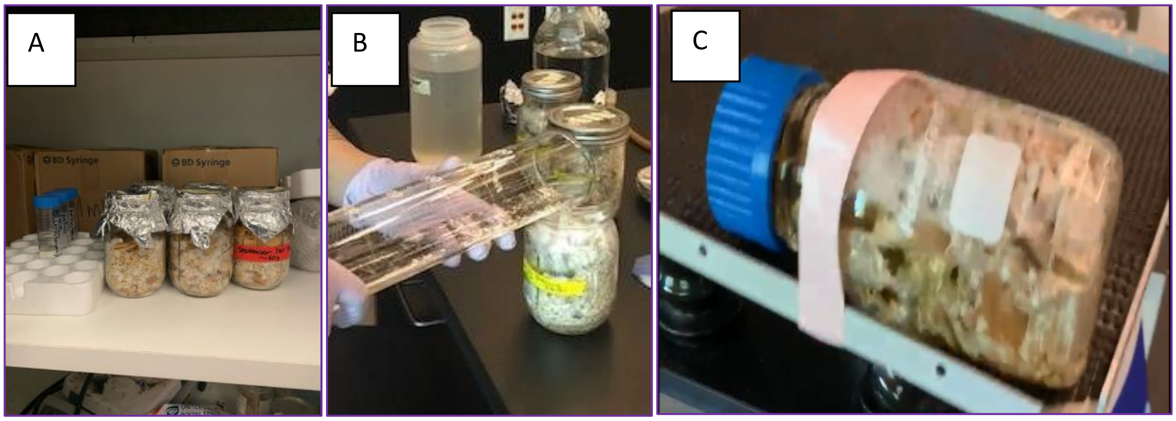

Figure S1 A: Three *Stropharia* biofilter columns on ring stands along with the ‘control’ containing uninoculated wood chips. Wetland water was run through each column. Each column had water held for a specific length of time, before samples were collected. Triplicate samples were collected

at each time point. **B: Frame A.** Three filter jars colonized with *S. rugosoannulata*, *P. ostreatus*, *P. pulmonarius*. **Frame B.** Water,170 -180 ml being added to the jar. **Frame C.** Jar shaken horizontally on a shaker at 120 RPM/minute.

Figure S2: Flowchart of Antibiotic resistance studies:

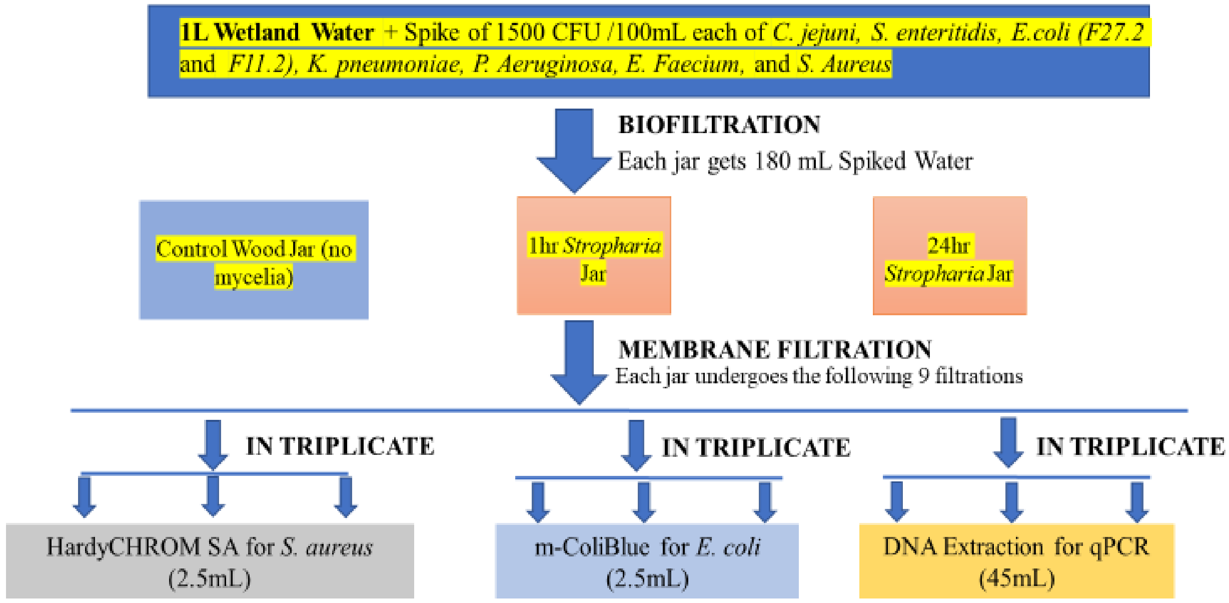

Figure S3

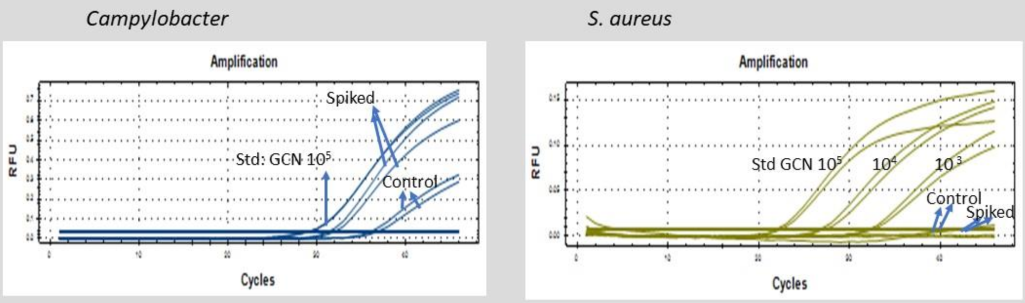

Campylobacter

Well	Fluor	Target	Content	Sample	Cq	SQ
A04	FAM		Unkn	stroph cont.	36.07	1.06E+03
B04	FAM		Unkn	stroph cont.	36.69	6.96E+02
G03	FAM		Unkn	spike stro	31.26	2.83E+04
H03	FAM		Unkn	spike stro	31.95	1.77E+04
G01	FAM		Std		29.08	1.00E+05
H01	FAM		Std		28.94	1.00E+05

S. aureus

Well	Fluor	Target	Content	Sample	Cq	SQ
A04	VIC		Unkn	stroph cont.	N/A	N/A
B04	VIC		Unkn	stroph cont.	41.1	1.65E+01
G03	VIC		Unkn	spike stro	42.11	1.05E+01
H03	VIC		Unkn	spike stro	N/A	N/A
C01	VIC		Std		31.99	1.00E+03
D01	VIC		Std		32.33	1.00E+03
E01	VIC		Std		26.95	1.00E+04
F01	VIC		Std		26.55	1.00E+04
G01	VIC		Std		21.75	1.00E+05
H01	VIC		Std		22.26	1.00E+05

Amplification curves from qPCR run showing capture of *Campylobacter* spp but not *S. aureus* by Stropharia mycelia: qPCR using primers and probes specific for *Campylobacter* spp. and *S. aureus*, respectively, were used in a duplex reaction with 7 µl DNA extracts from control and spiked sample of mycelia in a final PCR volume of 20 µl. Three standards were included in each run and they were 10 ^{3}, 10^4, 10 ^{5} GCN of a pure preparation of *S. aureus* and *C. jejuni* genomic DNA. In the figure "Campylobacter" only one standard has been shown.

Table S1: **Bacterial Strains and Growth Media**

**Bacterial Strain**	**Isolate Number**	**Growth Media**	**Resistance Genes**	**Size of genome used to calculate (GCN)**	**Reference**
Escherichia coli	F11.2	Luria Broth + tetracycline + cefotaxime;<br>-<br>m-coli blue24 broth (Hach)	*bla*$_{CTX}$, *tet* (M), *tet*(A), *tet* (B) *strA, sul1*	-	Sen et al (2019)
Escherichia coli	F42.2	Luria Broth + ampicillin;<br><br>m-coli blue broth24 (Hach)	*bla*$_{CMY,}$ *strB*	-	Sen et al (2019)
Campylobacter jejuni	F39.2	Campy CVA	*tet*(O)	2.5 Mb	Sen et al (2022)
Salmonella enteritidis	ATCC 13076	Xylose Lysine Deoxycholate (XLD) Agar		5.027 Mb	
Enterococcus faecium Van ^{R}	ATCC 700221	Bile Esculin Agar (BEA) or Columbia Blood Agar	*vanA*	2.9 Mb	
Pseudomonas aeruginosa	ATCC 27583	M-PA-C Agar		6.84 Mb	
Klebsiella pneumoniae	ATCC BA 2146	Eosin methylene blue (EMB) Agar		5.491 Mb	
Staphylococcus Aureus	ATCC 25923	Mannitol Salt Agar ; Chromagar SA (Hardy diagnostics)		-	

Table S2: Table of Primers

Target gene	Primer name	Primer sequence (5' – 3')	Amplicon size (bp)	References
strA	strA-F	TCAATCCCGACTTCTTACCG	126	Walsh, et al. (2011)
	strA-R	CACCATGGCAAACAACCATA		
	strA-Probe	HEX-TGCTCGACCAAGAGCGGC-BHQ-1		
strB	strB-F	ATCGCTTTGCAGCTTTGTTT	143	Walsh, et al. (2011)
	strB-R	ATGATGCAGATCGCCATGTA		
	strB-Probe	FAM-ATGCCTCGGAACTGCGT-BHQ-2		
sul 1	FP_Sul1_2	ACGAGATTGTGCGGTTCTTC	159	Schmidt, et al. (2015) [10]
	RP_Sul1_2	CCGACTTCAGCTTTTGAAGG		
	PR_Sul1_2	ACCGGCTCATCCTCGATCCG		
***tet* (A)**	FP TETA 2	TTGGCATTCTGCATTCACTC -		
	RP_TETA_2	GAAGGCAAGCAGGATGTAGC	125 bp	Schmidt, et al. (2015) [10]
	PR_TETA_2	GATCACCGGCCCTGTAGCCG		

***tet* (B)**		(Proprietary assay from Applied Biosystems)		Catalog # 1 xNC1998976, Ba04932083_s1
***tet* (M)**	tet (M)-F	GGTTTCTCTTGGATACTTAA ATCAATCR	67	Walsh, et al. (2011) [12]
	tet (M)-R	CCAACCATAYAATCCTTGTT CRC		
	tet (M)-Probe	FAM=ATGCAGTTATGGARGG GATACGCTATGGY-BHQ-1		
***tet* (O)**	FP_TETO_ Böck	AAGAAAACAGGAGATTCCA AAACG		Böckelmann, et al. (2008)
	RP_TETO_ Böck	CGAGTCCCCAGATTGTTTTT AGC		
		SUN-ACGTTATTTCCCGTTTATCAC GGAAGCG-BHQ-1		
bla-CMY-2	FW3_CMY-2_Lahey	AGACGTTTAACGGCGTGTTG	127	Schmidt, et al. (2015)
	RV4_CMY-2_Lahey C	TAAGTGCAGCAGGCGGATA		
	PR_CMY-2_Lahey	TATCGCCCGCGGCGAAAT		
CTX-M	**consensus primer -F**	ATGTGCAGYACCAGTAARGT KATGGC		Birkett, et al. (2007)
	**consensus primer-R**	ATCACKCGGRTCGCCXGG RAT		
	**CTX-M-1 group probe(FAM)**	CAGGTGCTTATCGCTCTCGC TCTGTT-BHQ		
	**CTX-M probe for all groups W/O**	CGACAATACNGCCATGAA-MGB-NFQ		

	**CTX-M-1(VIC)**			
vanA	FP_vanA_	CTGTGAGGTCGGTTGTGCG	64	Böckelmann, et al. (2008)
	RP_vanA_	TTTGGTCCACCTCGCCA 60		
	PR_vanA_	CAACTAACGCGGCACTGTTTCCCAAT		
Salmonella (invA)	invA_176F	CAACGTTTCCTGCGGTACTGT	116	Gonzalez-Escalona, et al. (2009)
	invA_291R	CCCGAACGTGGCGATAATT		
	invA-Tx_209(FAM)	CTCTTTCGTCTGGCATTATCGATCAGTACCA-TAMRA		
Campylobacter species (16S rRNA)	Camp-F3	CACGTGCTACAATGGCATAT	108	Lund, et al. (2004)
	CampR3	GGCTTCATGCTCTCGAGTT		
	Camp-P2 (Probe_FAM)	CAG AGA ACA ATC CGA ACT GGG ACA BHQ1		
Staphylococcus aureus (ebpS)	868F	CCACATGCCTCTAATAATG	197	Liu, et al. (2019)
	1064R	GCGATTTTATTTTCTTTTGTAC		
	1024P (Probe_VIC)	ATGCCATGCCTCCAAATATCGC		
Pseudomonas aeruginosa	gyrB418F	AACAAGGTCTGGGAACAGGTCTAC		Golpayegani, et al. (2019)

(gyrB)	gyrB490R	CATCGG TCTCGCCCACTTC		
	gyrB444P (Probe_FAM)	CCACGGCGTTCCGCAGTTCC-BHQ2		
E. faecium (23S rRNA)	EnteroF1A	GAG AAA TTC CAA ACG AAC TTG	92	Ryu,etal. (2012)
	ENC854R	CAG TGC TCT ACC TCC ATC ATT		
	GPL813TQ	VIC-TGG TTC TCT CCG AAA TAG CTT TAG GGC TA-TAMRA		
S. aureus				
E. coli				
***K.pneumoniae* Bacterial (16S rDNA)**	ABI	Proprietary assay from Applied Biosystems)	466	Catalog # 1 x NC1998976, Ba04932083
	16S rDNA-F	TCCTACGGGAGGCAGCAGT		
	16S rDNA-R	GGACTACCAGGGTATCTAAT CCTGTT		Nadkarni et al (2002)
**Fungal ID-1 (ITS1-5.8S-ITS2)**	16S rDNA-Pr	FAM-CGTATTACCGCGGCTGCTGG CAC-BHQ-1 466		
**Fungal ID-1 (ITS1-5.8S-ITS2)**	**ITS-1-F**	CTTGGTCATTTAGAGGAAGT AA		Vlasenko, etal. (2019)
**Fungal ID-2**	**ITS4-B**	CAGGAGACTTGTACACGGT CCAG		

**(28S rRNA)**	**LRoR- LSU F**	GTACCCGCTGAACTTAAGC;		Eberhardt U (2012)
	**LR3- LSUR**	GGTCCGTGTTTCAAGAC		
